# A Comparative Study of Wireless Sensor Networks and Their Routing Protocols

**DOI:** 10.3390/s101210506

**Published:** 2010-11-24

**Authors:** Debnath Bhattacharyya, Tai-hoon Kim, Subhajit Pal

**Affiliations:** 1 Department of Multimedia Engineering, Hannam University, Daejeon, Korea; E-Mail: debnathb@gmail.com; 2 Heritage Institute of Technology, Kolkata-700107, India; E-Mail: pal.subhajit77@gmail.com

**Keywords:** wireless sensors, protocols, routing, energy efficiency, clustering

## Abstract

Recent developments in the area of micro-sensor devices have accelerated advances in the sensor networks field leading to many new protocols specifically designed for wireless sensor networks (WSNs). Wireless sensor networks with hundreds to thousands of sensor nodes can gather information from an unattended location and transmit the gathered data to a particular user, depending on the application. These sensor nodes have some constraints due to their limited energy, storage capacity and computing power. Data are routed from one node to other using different routing protocols. There are a number of routing protocols for wireless sensor networks. In this review article, we discuss the architecture of wireless sensor networks. Further, we categorize the routing protocols according to some key factors and summarize their mode of operation. Finally, we provide a comparative study on these various protocols.

## 1. Introduction

A wireless sensor network (WSN) consists of hundreds to thousands of low-power multi-functional sensor nodes, operating in an unattended environment, and having sensing, computation and communication capabilities. The basic components [1] of a node are a sensor unit, an ADC (Analog to Digital Converter), a CPU (Central processing unit), a power unit and a communication unit. Sensor nodes are micro-electro-mechanical systems [2] (MEMS) that produce a measurable response to a change in some physical condition like temperature and pressure. Sensor nodes sense or measure physical data of the area to be monitored. The continual analog signal sensed by the sensors is digitized by an analog-to-digital converter and sent to controllers for further processing. Sensor nodes are of very small size, consume extremely low energy, are operated in high volumetric densities, and can be autonomous and adaptive to the environment. The spatial density of sensor nodes in the field may be as high as 20 nodes/m^3^. As wireless sensor nodes are typically very small electronic devices, they can only be equipped with a limited power source [3]. Each sensor node has a certain area of coverage for which it can reliably and accurately report the particular quantity that it is observing. Several sources of power consumption in sensors are: (a) signal sampling and conversion of physical signals to electrical ones; (b) signal conditioning, and (c) analog-to-digital conversion.

There are three categories of sensor nodes:

- Passive, Omni Directional Sensors: passive sensor nodes sense the environment without manipulating it by active probing. In this case, the energy is needed only to amplify their analog signals. There is no notion of “direction” in measuring the environment.
- Passive, narrow-beam sensors: these sensors are passive and they are concerned about the direction when sensing the environment.
- Active Sensors: these sensors actively probe the environment.

Since a sensor node has limited sensing and computation capacities, communication performance and power, a large number of sensor devices are distributed over an area of interest for collecting information (temperature, humidity, motion detection, *etc.*). These nodes can communicate with each other for sending or getting information either directly or through other intermediate nodes and thus form a network, so each node in a sensor network acts as a router [4] inside the network. In direct communication routing protocols (single hop), each sensor node communicates directly with a control center called Base Station (BS) and sends gathered information. The base station is fixed and located far away from the sensors. Base station(s) can communicate with the end user either directly or through some existing wired network. The topology of the sensor network changes very frequently. Nodes may not have global identification. Since the distance between the sensor nodes and base station in case of direct communication is large, they consume energy quickly. In another approach (multi hop), data is routed via intermediate nodes to the base station and thus saves sending node energy. A routing protocol [5] is a protocol that specifies how routers (sensor nodes) communicate with each other, disseminating information that enables them to select routes between any two nodes on the network, the choice of the route being done by routing algorithms. Each router has *a priori* knowledge only of the networks attached to it directly. A routing protocol shares this information first among immediate neighbors, and then throughout the network. This way, routers gain knowledge of the topology of the network. There are mainly two types of routing process: one is static routing and the other is dynamic routing.

Dynamic routing [6] performs the same function as static routing except it is more robust. Static routing allows routing tables in specific routers to be set up in a static manner so network routes for packets are set. If a router on the route goes down, the destination may become unreachable. Dynamic routing allows routing tables in routers to change as the possible routes change. In case of wireless sensor networks dynamic routing is employed because nodes may frequently change their position and die at any moment. The advantages and disadvantages of wireless sensor networks can be summarized as follows:

Advantages:

- Network setups can be done without fixed infrastructure.
- Ideal for the non-reachable places such as across the sea, mountains, rural areas or deep forests.
- Flexible if there is *ad hoc* situation when additional workstation is required.
- Implementation cost is cheap.

Disadvantages:

- Less secure because hackers can enter the access point and get all the information.
- Lower speed compared to a wired network.
- More complex to configure than a wired network.
- Easily affected by surroundings (walls, microwavea, large distances due to signal attenuation, *etc.*).

**Figure 1.**
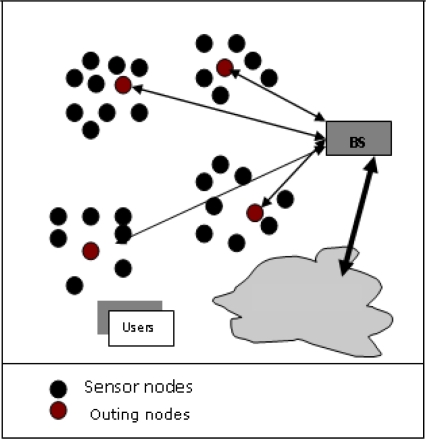
A wireless sensor network structure.

A Wireless Sensor Network structure is shown in Figure 1.

## 2. Recent Works

Martin Merck [36], in 2010, in his paper has described function of IceCube installed at South Pole. As per his observation IceCube is the largest Neutrino observatory currently in operations. Located at the geographical South Pole, the detector modules are deployed up to 2,450 m deep into the Antarctic ice. A combination of intelligent sensor modules and a farm of industry standard servers are used to operate the detector and reduce the data to accommodate the limited connectivity from the South Pole to the northern hemisphere. He has given a detailed description of the technical implementation of the sensor modules, data acquisition system and filtering farm used in the IceCube experiment.

You-Chiun Wang, *et al*, in March 2010, considered a hybrid wireless sensor network with static and mobile nodes. Static sensors monitor the environment and report events occurring in the sensing field. They scheduled the mobile sensors’ traveling paths in an energy-balanced way so that their overall lifetime could be maximized and they shown that it has been a NP-complete problem. They proposed a centralized and a distributed heuristics to schedule mobile sensors' traveling paths. Their heuristics allowed arbitrary numbers of mobile sensors and event locations in each round and had an energy-balanced concept in mind. The centralized heuristic tries to minimize mobile sensors’ moving energy while keeping their energy consumption balanced [37].

Kunjan Patel, *et al.*, presented a reliable and lightweight routing protocol for wireless sensor networks in their paper. They claimed more than 90% savings in number of transmissions compared to the message flooding scheme when the same route was used to transmit data messages. This saving increased exponentially as the number of transmissions increased over a same route. The protocol occupied only 16% of total available RAM and 12% of total program memory in MICAz platform which maked it very lightweight to implement in wireless sensor networks [38].

Mohamed Hafeeda and Hossein Ahmadi, 2007, proposed [39] a new probabilistic coverage protocol (denoted by PCP) that considered probabilistic sensing models. PCP was fairly general and used with different sensing models. In particular, PCP required the computation of a single parameter from the adopted sensing model, while everything else remained same. They showed how this parameter could be derived in general, and the calculations for two example sensing models: (i) the probabilistic exponential sensing model, and (ii) the commonly-used deterministic disk sensing model. They compared their protocol with two existing protocols and claimed for the better performance as they proposed.

## 3. Applications

The applications for WSNs involve tracking, monitoring and controlling. WSNs are mainly utilized for habitat monitoring, object tracking, nuclear reactor control, fire detection, and traffic monitoring. Area monitoring is a common application of WSNs, in which the WSN is deployed over a region where some incident is to be monitored. For example, a large quantity of sensor nodes could be deployed over a battlefield to detect enemy intrusions instead of using landmines. When the sensors detect the event being monitored (heat, pressure, sound, light, electro-magnetic field, vibration, *etc.*), the event needs to be reported to one of the base stations, which can than take some appropriate action (e.g., send a message on the internet or to a satellite). Wireless sensor networks are used extensively within the water/wastewater industries. Facilities not wired for power or data transmission can be monitored using industrial wireless I/O devices and sensor nodes powered by solar panels or battery packs. Wireless sensor networks can use a range of sensors to detect the presence of vehicles for vehicles detection. Wireless sensor networks are also used to control the temperature and humidity levels inside commercial greenhouses. When the temperature and humidity drops below specific levels, the greenhouse manager can be notified via e-mail or a cell phone text message, or host systems can trigger misting systems, open vents, turn on fans, or control a wide variety of system responses. Because some wireless sensor networks are easy to install, they are also easy to move when the needs of the application change.

## 4. Classification

Routing techniques are required for sending data between sensor nodes and the base stations for communication. Different routing protocols are proposed for wireless sensor network. These protocols are classified according to different parameters. Protocols can be classified as proactive, reactive and hybrid, based on their mode of functioning and type of target applications. In a proactive protocol the nodes switch on their sensors and transmitters, sense the environment and transmit the data to a BS through the predefined route. The Low Energy Adaptive Clustering hierarchy protocol (LEACH) utilizes this type of protocol [7]. In case of a reactive protocol if there are sudden changes in the sensed attribute beyond some pre-determined threshold value, the nodes immediately react. This type of protocol is used in time critical applications. The Threshold sensitive Energy Efficient sensor Network (TEEN) [8] is an example of a reactive protocol. Hybrid protocols like Adaptive Periodic TEEN (APTEEN) incorporate both proactive and reactive concepts [9]. They first compute all routes and then improve the routes at the time of routing. Further, routing protocols can be classified as direct communication, flat and clustering protocols, according to the participation style of the nodes. In direct communication protocols, any node can send information to the BS directly. When this is applied in a very large network, the energy of sensor nodes may be drained quickly. Its scalability is very small. SPIN is an example of this type of protocol. In the case of flat protocols, for example Rumor Routing, if any node needs to transmit data, it first searches for a valid route to the BS and then transmits the data. Nodes around the base station may drain their energy quickly. Its scalability is average. According to the clustering protocol, the total area is divided into numbers of clusters. Each and every cluster has a cluster head (CH) and this cluster head directly communicates with the BS. All nodes in a cluster send their data to their corresponding CH (example: TEEN). Furthermore, depending on the network structure, protocols can be classified as hierarchical, data centric and location based. Hierarchical routing (examples: LEACH, TEEN, APTEEN) is used to perform energy efficient routing, *i.e.*, higher energy nodes can be used to process and send the information; low energy nodes are used to perform the sensing in the area of interest. Data centric protocols are query based and they depend on the naming of the desired data, thus it eliminates much redundant transmissions. The BS sends queries to a certain area for information and waits for reply from the nodes of that particular region. Since data is requested through queries, attribute based naming is required to specify the properties of the data. Depending on the query, sensors collect a particular data from the area of interest and this particular information is only required to transmit to the BS and thus reducing the number of transmissions. SPIN [10] was the first data centric protocol. Location based routing protocols [11] need some location information of the sensor nodes. Location information can be obtained from GPS (Global Positioning System) signals, received radio signal strength, *etc.* Using location information, an optimal path can be formed without using flooding techniques. GEAR is an example of a location based routing protocol. The present review discusses the intricate details of the roles of different routing protocols. Furthermore it provides a comparative analysis between these.

## 5. Sensor Network Architecture and Design Issues

The main design goal of wireless sensor networks is to transmit data by increasing the lifetime of the network and by employing energy efficient routing protocols. Depending on the applications used, different architectures and designs have been applied in sensor networks. Again, the performance of a routing protocol depends on the architecture and design of the network, so the architecture and design of the network is very important features in WSNs. The design of the wireless sensor network is affected by many challenging factors which must be overcome before an efficient network can be achieved in WSNs. In the following section we try to describe the architectural issues and challenges for WSNs.

Node Distribution: Node distribution [12] in WSNs is either deterministic or self-organizing and application dependant. The uniformity of the node distribution directly affects the performance of the routing protocol used for this network. In the case of deterministic node distribution, the sensor nodes are mutually placed and gathered data is transmitted through pre-determined paths. In the other case, the sensor nodes are spread over the area of interest randomly thus creating an infrastructure in an *ad hoc* manner.

Network Dynamicity: Since the nodes in WSNs may be static or dynamic, dynamicity of the network is a challenging issue. Most of the routing protocols assume that the sensor nodes and the base stations are fixed *i.e*., they are static, but in the case of dynamic BS or nodes routes from one node to another must be reported periodically within the network so that all nodes can transmit data via the reported route. Again depending on the application, the sensed event can be dynamic or static. For example, in target detection/tracking applications, the event is dynamic, whereas forest monitoring for early fire prevention is an example of a static event. Monitoring static events works in reactive mode. On the other hand, dynamic events work in proactive mode.

Energy efficiency: The sensor nodes in WSNs have limited energy and they use their energy for computation, communication and sensing, so energy consumption is an important issue in WSNs. According to some routing protocols nodes take part in data fusion and expend more energy. Since the transmission power is proportional to distance squared, multi-hop routing consumes less energy than direct communication, but it has some route management overhead. In this regard, direct communication is efficient. Since most of the times sensor nodes are distributed randomly, multi-hop routing is preferable. In some applications nodes sense environment periodically and lose more energy than the nodes used in some applications where they sense environment when some event occurs.

Data Transmission: Data transmission in WSNs is application specific. It may be continuous or event driven or query-based or hybrid. In case of continuous data transmission, sensor nodes send data to the base station periodically. In event driven and query-based transmission they send data to the base station when some event occurs or a specific query is generated by the base station. Hybrid transmission uses a combination of continuous, event driven and query-based transmission, so for architecture and design of WSNs data transmission is a very significant issue.

Scalability: A WSN consists of hundreds to thousands of sensor nodes. Routing protocols must be workable with this huge number of nodes *i.e.*, these protocols can be able to handle all of the functionalities of the sensor nodes so that the lifetime of the network can be stable.

Data Fusion: Data fusion [13] is a process of combining of data from different sources according to some function. This is achieved by signal processing methods. This technique is used by some routing protocols for energy efficiency and data transfer optimization. Since sensor nodes get data from multiple nodes, similar packets may be fused generating redundant data. In data fusion or data aggregation process awareness is needed to avoid this redundant data.

## 6. Existing Routing Protocols

### 6.1. LEACH (Low Energy Adaptive Clustering Hierarchy)

LEACH [7] is a self-organizing, adaptive clustering protocol. It uses randomization for distributing the energy load among the sensors in the network. The following are the assumptions made in the LEACH protocol:

- All nodes can transmit with enough power to reach the base station.
- Each node has enough computational power to support different MAC protocols.
- Nodes located close to each other have correlated data.

According to this protocol, the base station is fixed and located far from the sensor nodes and the nodes are homogeneous and energy constrained. Here, one node called cluster-head (CH) acts as the local base station. LEACH randomly rotates the high-energy cluster-head so that the activities are equally shared among the sensors and the sensors consume battery power equally. LEACH also performs data fusion, *i.e.* compression of data when data is sent from the clusters to the base station thus reducing energy dissipation and enhancing system lifetime. LEACH divides the total operation into rounds—each round consisting of two phases: set-up phase and steady phase.

In the set-up phase, clusters are formed and a CH is selected for each cluster. The CH is selected from the sensor nodes at a time with a certain probability. Each node generates a random number from 0 to 1. If this number is lower than the threshold node [T(n)] then this particular node becomes a CH. T(n) is given as follows:

$$T(n)=\frac{p}{1-p}\left[r\text{mod}\left(1/p\right)\right],\;n\in G=0,\;\text{otherwise}$$

where p is the percentage of nodes that are CHs, r is the current round and G is the set of nodes that have not served as cluster head in the past 1/p rounds.

**Figure 2.**
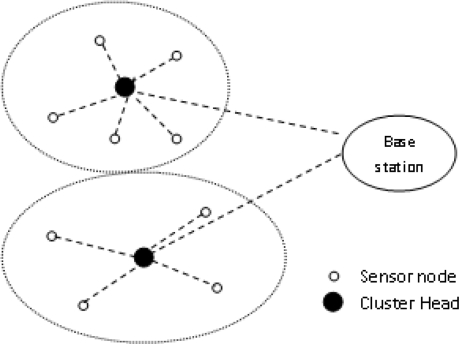
Clustering in LEACH Protocol.

Then the CH allocates time slots to nodes within its cluster. LEACH clustering is shown in Figure 2.

In steady state phase, nodes send data to their CH during their allocated time slot using TDMA. When the cluster head gets data from its cluster, it aggregates the data and sends the compressed data to the BS. Since the BS is far away from the CH, it needs high energy for transmitting the data. This affects only the nodes which are CHs and that’s why the selection of a CH depends on the remaining energy of that node.

### 6.2. TEEN (Threshold sensitive Energy Efficient sensor Network)

TEEN [8] is a cluster based hierarchical routing protocol based on LEACH. This protocol is used for time-critical applications. It has two assumptions [14]:

- The BS and the sensor nodes have same initial energy
- The BS can transmit data to all nodes in the network directly.

In this protocol, nodes sense the medium continuously, but the data transmission is done less frequently. The network consists of simple nodes, first-level cluster heads and second-level cluster heads. TEEN uses LEACH’s strategy to form cluster. First level CHs are formed away from the BS and second level cluster heads are formed near to the BS.

A CH sends two types of data to its neighbors—one is the hard threshold (HT) and other is soft threshold (ST). In the hard threshold, the nodes transmit data if the sensed attribute is in the range of interest and thus it reduces the number of transmissions. On the other hand, in soft threshold mode, any small change in the value of the sensed attribute is transmitted. The nodes sense their environment continuously and store the sensed value for transmission. Thereafter the node transmits the sensed value if one of the following conditions satisfied:

- Sensed value > hard threshold (HT).
- Sensed value ∼ hard threshold >= soft threshold (ST).

TEEN has the following drawbacks:

- A node may wait for their time slot for data transmission. Again time slot may be wasted if a node has no data for transmission.
- Cluster heads always wait for data from nodes by keeping its transmitter on.

### 6.3. APTEEN (Adaptive Threshold TEEN)

APTEEN [9] is an improved version of TEEN which has all the features of TEEN. It was developed for hybrid networks and captures both periodic data collection and reacts to time critical events. APTEEN supports queries like:

- Historical analysis of past data values
- A snapshot of the current network view.
- Persistent monitoring of an event for a period of time.

In each round, after deciding the cluster head, the cluster head broadcasts the following parameters:

- Attributes (interested physical parameters),
- Thresholds (hard threshold value and soft threshold value),
- time schedule (time slot using TDMA) and
- count time (maximum time period between two successive reports sent by a node).

**Figure 3.**
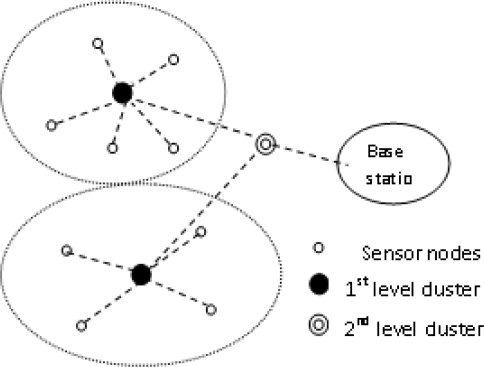
Hierarchical clustering in TEEN and APTEEN Protocols.

It allows the user to set threshold values and also a count time interval. If a node does not send data for a time period equal to the count time, it is forced to sense and retransmit the data thus maintaining energy consumption. Since it is a hybrid protocol, it can emulate a proactive network or a reactive network depending on the count time and threshold value. Figure 3 shows TEEN and APTEEN. It has the disadvantage that additional complexity is required to implement the threshold function and count time features.

### 6.4. PEGASIS (Power efficient Gathering Sensor Information System)

PEGASIS [15] is a near optimal chain-based power efficient protocol based on LEACH [7]. According to this protocol, all the nodes have information about all other nodes and each has the capability of transmitting data to the base station directly. PEGASIS assumes that all the sensor nodes have the same level of energy and they are likely to die at the same time. Since all nodes are immobile and have global knowledge of the network, the chain can be constructed easily by using greedy algorithm. Chain creation is started at a node far from BS. Each node transmits and receives data from only one closest node of its neighbors. To locate the closest neighbor node, each node uses the signal strength to measure the distance from the neighbors and then adjusts the signal strength so the only one node cab is heard. Node passes token through the chain to leader from both sides. Each node fuses the received data with their own data at the time of constructing the chain. In each round, a randomly chosen node (leader) from the chain will transmit the aggregated data to the BS. Node i (mod N) is the leader in round i. The chain consists of those nodes that are closest to each other and form a path to the base station. The aggregated data is sent to the base station by the leader.

**Figure 4.**
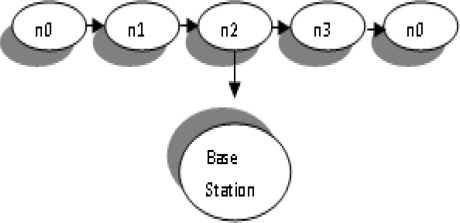
Chaining in PEGASIS.

PEGASIS outperforms LEACH by eliminating the overhead of dynamic cluster information, minimizes the sum of distances and limits the number of transmission. Each node requires global information about the network. This is a drawback of this protocol because at any time it can be collected from the network. PEGASIS is shown in Figure 4.

### 6.5. SPIN (Sensor Protocols for Information via Negotiation)

SPIN [16,17] is a family of adaptive protocols that use data negotiation and resource-adaptive algorithms. SPIN is a data centric routing protocol. It assumes:

- all nodes in the network are base stations.
- nodes in close proximity have similar data.

**Figure 5.**
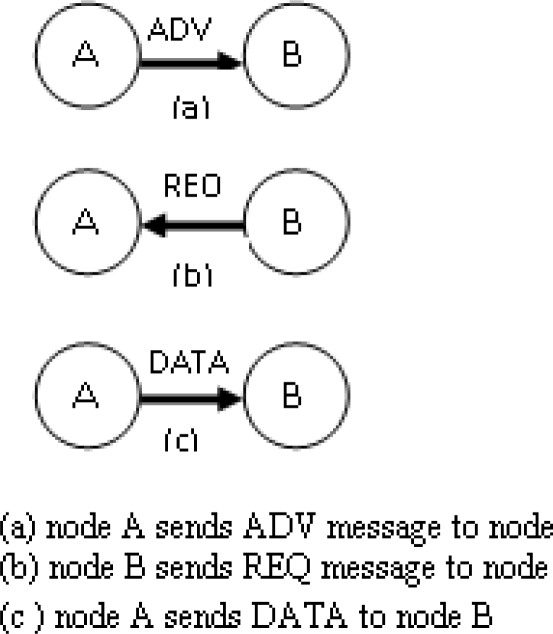
Data Transmission in SPIN.

The key idea behind SPIN is to name the data using high-level descriptors or meta-data. Since all nodes can be assumed as base stations all information is broadcasted to each node in the network. So user can query to any node and can get the information immediately. Nodes in this network use a high level name to describe their collected data called meta-data. Figure 5 shows how SPIN works.

Before transmission, meta-data are exchanged among sensors nodes (meta-data negotiation) via a data advertisement procedure, thus avoiding transmission of redundant data in the network. After receiving the data each node advertises it to its neighbors and interested neighbors get this data by sending a request message. The format of this meta-data is not specified in SPIN and it depends on the used applications. This meta-data negotiation solves the classic problem of flooding and thus it achieves energy efficiency. SPIN uses three types of messages: ADV, REQ, and DATA for communication with each other. ADV is used for adverting new data, REQ is used for requesting for data and DATA is the actual message. According to this protocol first a node gets some new data and the node wants to distribute that data throughout the network, so it broadcasts an ADV message containing meta-data. The interested nodes request that data by sending a REQ message and the data is sent to the requesting nodes.

The neighboring node repeats this process until the entire network gets the new data. The SPIN protocols include many other protocols. The main two protocols are SPIN-1 and SPIN-2. These two protocols incorporate negotiation before transmitting data so that only useful information will be transferred. Each node has its own resource manager that keeps track of resource consumption. The SPIN-1 protocol is a 3-stage protocol, as described above. SPIN-2 is an extension of SPIN-1, which incorporates threshold-based resource awareness mechanism in addition to negotiation. When energy in the nodes is abundant, SPIN-2 communicates using the 3-stage protocol of SPIN-1.

One of the advantages of SPIN is that topological changes are localized since each node only needs to know its single-hop neighbors. SPIN provides much more energy savings than flooding and meta-data negotiation almost halves the redundant data. However, SPINs data advertisement mechanism cannot guarantee the delivery of data. To see this, consider the application of intrusion detection where data should be reliably reported over periodic intervals and assume that nodes interested in the data are located far away from the source node and the nodes between source and destination nodes are not interested in that data, such data will not be delivered to the destination at all.

### 6.6. DD (Directed Diffusion)

**Figure 6.**
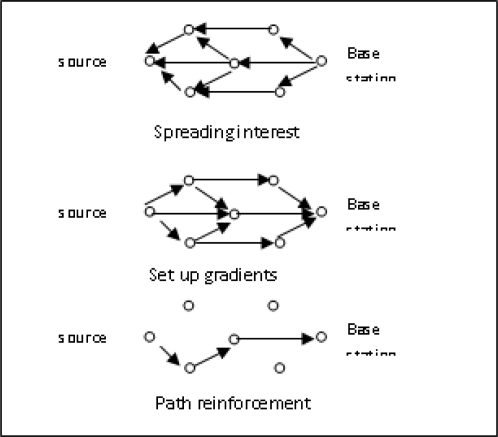
Directed Diffusion Protocol.

Directed diffusion [17,18] is a data-centric (DC) and application-aware protocol in which data generated by sensor nodes is named by attribute-value pairs. It consists of four elements: [14] interests, data messages, gradients and reinforcements. An interest (a list of attribute value pairs) describes a task. Data messages are named using attribute value pairs. A gradient specifies data rate as well as the direction of event and reinforcement selects a particular path from a number of paths. In the DC protocol data coming from different sources are combined and thus eliminating redundancy, minimizing the number of transmissions, saving network energy and prolonging its lifetime. DC routing searches for a destination from multiple sources. In directed diffusion, a base station diffuses a query towards nodes in the interested region. The query or interest is diffused through the network hop-by-hop. Each sensor receives the interest and sets up a gradient toward the sensor nodes from which it receives the interest. This process continues until gradients are set up from the sources back to the BS. The sensed data are then returned to the BS along that reverse path. The intermediate nodes may aggregate their data depending on the data message (data’s name and attribute value pair) thus reducing the communication cost. Since in this case data transmission is not reliable the BS periodically refreshes and resends the interest when it starts to receive data from the source(s). Directed Diffusion protocols are application specific and hence can save energy by selecting optimal paths by caching and processing data in the network. It has some drawbacks [14]. First of all, for data aggregation it needs time synchronization technique that is not very easy to achieve in WSNs. Another problem is associated with the overhead involved in recording information thus increasing the cost of a sensor node. The DD Protocol is described in Figure 6.

### 6.7. Rumor Routing

Rumor routing [19,20] is a kind of directed diffusion and is used for applications where geographic routing is not feasible. It combines query flooding and event flooding protocols in a random way. It has the following assumptions:

- The network is composed of densely distributed nodes.
- Only bi-directional links exits.
- Only short distance transmissions are allowed.
- It has fixed infrastructure.

In case of directed diffusion flooding is used to inject the query to the entire network. Sometimes the requested data from the nodes are very small and thus the flooding is unnecessary, so we can use another approach which is to flood the events when the number of events is small and the number of queries is large. The queries are rooted to that particular nodes that are belongs to the interested region. In order to flood events through the network, the rumor routing algorithm employs long-lived packets, called agents. When a node detects an event, it adds such event to its local table (events table), and generates an agent. Agents travel the network on a random path with related event information. Then the visited nodes form a gradient towards the event. When a node needs to initiate a query, it routes the query to the initial source. If it gets some nodes lying on the gradient before its TTL expires, it will be routed to the event, else the node may need to retransmit, give up or flood the query. Unlike directed diffusion, where data can be routed through multiple paths at low rates, Rumor routing only maintains one path between source and destination. Rumor routing performs well only when the number of events is small. For a large number of events, the cost of maintaining agents and event-tables in each node becomes infeasible if there is not enough interest in these events from the BS. Moreover, the overhead associated with rumor routing is controlled by different parameters used in the algorithm such as time-to-live (TTL) pertaining to queries and agents.

### 6.8. Geographic and Energy-Aware Routing (GEAR)

Location based routing protocols for sensor network need location information of all the sensor nodes to calculate the distance between any two nodes. GEAR [17,21] is a location based routing protocol which uses GIS (Geographical Information System) to find the location of sensor nodes in the network. According to this protocol, each node stores two types of cost of reaching the destination: estimated cost and learning cost. The estimated cost is a combination of residual energy [22] and distance to destination. The learned cost is a modified estimated cost and it accounts the routing around holes in the network. When a node does not have any closure neighbours towards the target region, a hole occurs. In case where no holes exit, the estimated cost is equal to the learned cost. The GEAR protocol only considers a certain region rather than sending the interests to the whole network as happens in Directed Diffusion [18] and thus restricting the number of interests. There are two phases in this protocol:

Phase-I: In this phase, packets are forwarded towards the target region. After receiving a packet, a node searches for a neighbor which is closer to the target region then itself. The neighbor is then selected as the next hop. If there are more than one suitable nodes then there exists a hole and in this case one node is picked to forward the packet based on the learning cost function.

Phase-II: In this phase, the packets are forwarded within the region. If the packet reaches the region, it is diffused in that region by either recursive geographic forwarding or restricted flooding. If the sensors are not densely deployed, then restricted flooding is used and if the node density is high, then geographic flooding is used. In geographic flooding, the region is divided into four sub regions and four copies of the packet are created. This process continues until the regions with only one node are left.

### 6.9. Geographic Adaptive Fidelity (GAF)

GAF is an energy efficient location-based routing protocol. This protocol was initially conceived for mobile *ad hoc* networks, but it can also be applied to sensor networks. GAF can be implemented both for non-mobile and mobile nodes. Although GAF is a location based protocol, it may also be implemented as a hierarchical protocol where the clusters are based on geographic location.

Initially the area of interest is split into some fixed zones forming a virtual grid for the covered area. Nodes in each zone have different functionalities and each node uses its GPS-indicated location to associate itself with a point in the grid. Nodes which are positioned at the same point on the grid are considered equivalent in terms of the cost of packet routing. Such equivalence is exploited in keeping some nodes located in a particular grid area in a sleeping state in order to save energy. Thus GAF can increase the network lifetime as the number of nodes increases. GAF conserves energy by turning off unnecessary nodes in the network without affecting the level of routing fidelity. GAF defines three states: discovery, active, sleep. The ‘discovery’ state is used for determining the neighbors in the grid; the ‘active’ state participates in routing process and at the time of ‘sleep’ state, the radio is turned off. In order to handle the mobility, each node in the grid estimates it’s leaving time of grid and sends this to its neighbors. The sleeping neighbors adjust their sleeping time accordingly in order to keep the routing fidelity. Before the leaving time of the active node expires, sleeping nodes wake up and one of them becomes active.

## 7. Comparative Study

Now we compare the above mentioned routing protocols according to their performance depending on different parameters. Table 1 shows the comparison.

LEACH, TEEN, APTEEN and PEGASIS have similar features and their architectures are to some extent similar. They have fixed infrastructure. LEACH, TEEN, APTEEN are cluster based routing protocols, whereas PEGASIS is a chain-based protocol. The performance of APTEEN lies between TEEN and LEACH with respect to energy consumption and longevity of the network [9]. TEEN only transmits time-critical data, while APTEEN performs periodic data transmissions. In this respect APTEEN is also better than LEACH because APTEEN transmits data based on a threshold value whereas LEACH transmits data continuously. Again PEGASIS avoids the formation of clustering overhead of LEACH, but it requires dynamic topology adjustment since sensor energy is not tracked. PEGASIS introduces excessive delay for distant nodes on the chain. The single leader can become a bottleneck in PEGASIS. PEGASIS increases network lifetime two-fold compared to the LEACH protocol.

In directed diffusion the base station sends queries to sensor nodes by the flooding technique but in SPIN the sensor nodes advertise the availability of data so that interested nodes can query that data. In Directed diffusion each node can communicate with its neighbors, so it does not need the total network information, but SPIN maintains a global network topology. SPIN halves the redundant data in comparison to flooding. Since SPIN cannot guarantee data delivery, it is not suitable for applications that need reliable data delivery.

SPIN, directed diffusion and rumor routing use meta-data whereas the other protocols don’t use it. Since they are flat routing protocols routes are formed in regions that have data for transmission, but for the others, as they are hierarchical routing methods they form clusters throughout the network. In case of hierarchical routing energy dissipation is uniform and it can’t be controlled; but in the case of flat routing energy dissipation depends on the traffic pattern. For the previous case data aggregation is done by cluster heads but in the later case, nodes on multi-hop path aggregates incoming data from neighbours. GEAR limits the number of interests in Directed Diffusion by considering only a certain region rather than sending the interests to the whole network. GEAR thus complements Directed Diffusion and conserves more energy. According to simulation results [17], GAF performs at least as well as a normal *ad hoc* routing protocol in terms of latency and packet loss and increases the lifetime of the network by saving energy. Since the sensor networks are application specific, we can’t say a particular protocol is better than other.

## 8. Conclusions

The past few years have witnessed a lot of attention on routing for wireless sensor networks and introduced unique challenges compared to traditional data routing in wired networks. Routing in sensor networks is a new area of research. Since sensor networks are designed for specific applications, designing efficient routing protocols for sensor networks is very important. In our work, first we have gone through a comprehensive survey of routing techniques in wireless sensor networks. The routing techniques are classified as proactive, reactive and hybrid, based on their mode of functioning and type of target applications. Further, these are classified as direct communication, flat and clustering protocols, according to the participating style of nodes. Again depending on the network structure, these are categorized as hierarchical, data centric and location based. In this document we have discussed eight routing protocols and their comprehensive survey in Section 2. These eight protocols are LEACH, TEEN, APTEEN, PEGASIS, SPIN, DD, RR and GEAR. Since the sensor networks are application specific, we can’t say any particular protocol is better than other. We can compare these protocols with respect to some parameters only. Future perspectives of this work are focused towards modifying one of the above routing protocols such that the modified protocol could minimize more energy for the entire system.

## Figures and Tables

**Table 1.** Comparison of different routing protocols.

**Protocols**	**Mobility**	**Power management**	**Network lifetime**	**Scalability**	**Resource awareness**	**Classification**	**Data aggregation**	**Query based**	**Multipath**
LEACH	Fixed BS	Maximum	Very good	Good	Yes	Clustering	No	No	No
TEEN	Fixed BS	Maximum	Very good	Good	Yes	Reactive/Clustering	Yes	No	No
APTEEN	Fixed BS	Maximum	Very good	Good	Yes	Hybrid	Yes	No	No
PEGASIS	Fixed BS	Maximum	Very good	Good	Yes	Reactive/Clustering	Yes	No	No
SPIN	Supported	Limited	Good	Limited	Yes	Proactive/flat	Yes	Yes	Yes
DD	Limited	Limited	Good	Limited	Yes	Proactive/flat	Yes	Yes	Yes
RR	Very limited	Not support	Very good	Good	Yes	Hybrid/flat	Yes	Yes	No
GEAR	Limited	Limited	Good	Limited	Yes	Location	No	No	No
GAF	Limited	Limited	Good	Limited	Yes	Location	No	No	No

BS: Base Station
